# Supplementary material for: Seminoma and Embryonal Carcinoma Footprints Identified by Analysis of Integrated Genome-Wide Epigenetic and Expression Profiles of Germ Cell Cancer Cell Lines
Source: PLoS One. 2014 Jun 2;9(6):e98330. doi: 10.1371/journal.pone.0098330 (PMC4041891; doi:10.1371/journal.pone.0098330)
Supplement: File S1 — ZIP file containing DMRforPairs output for significant regions. Please start from the html files. (ZIP) [file pone.0098330.s008.zip › figures/34618.pdf]

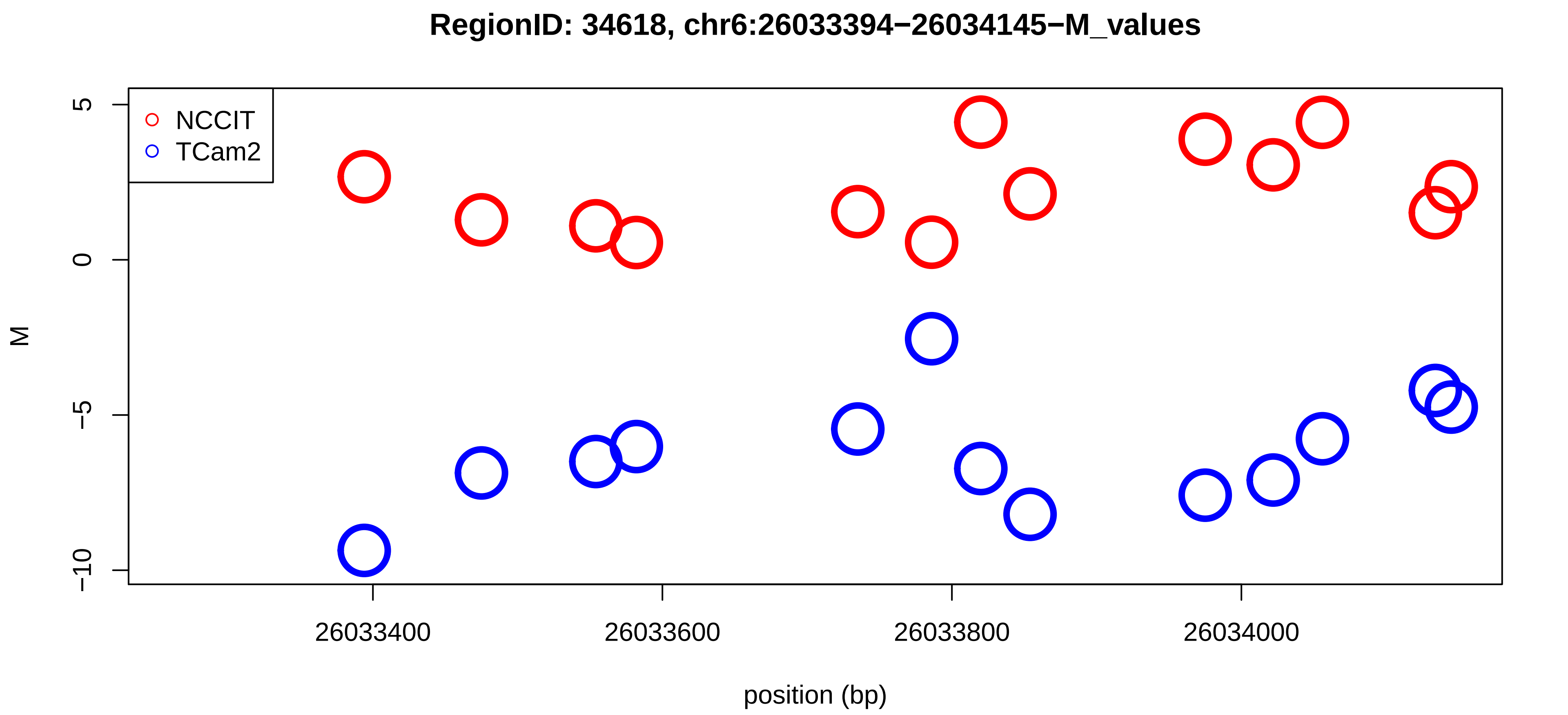
RegionID: 34618, chr6:26033394−26034145−M_values
NCCIT
TCam2
M
5
0
−5
−10
26033400
26033600
26033800
26034000
position (bp)

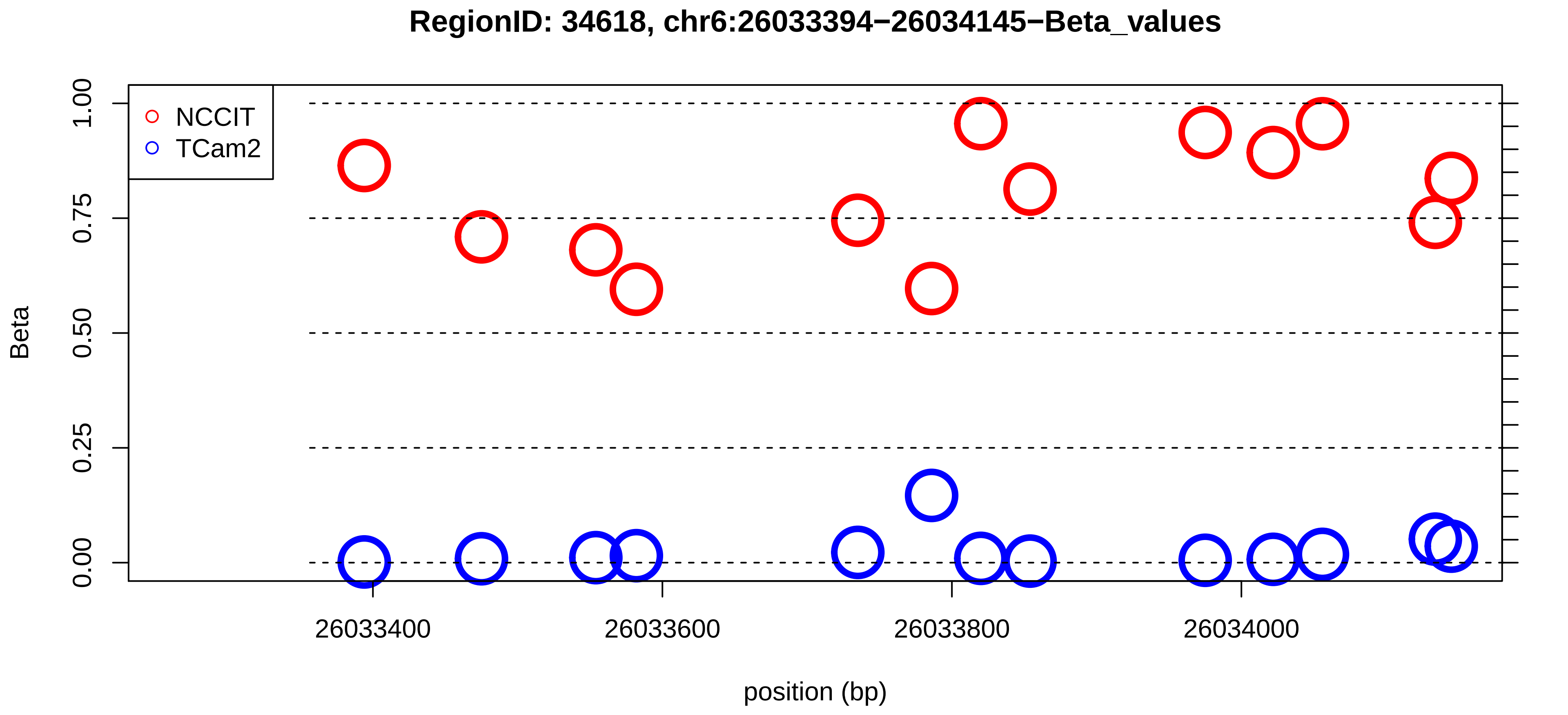
RegionID: 34618, chr6:26033394−26034145−Beta_values
NCCIT
TCam2
Beta
1.00
0.75
0.50
0.25
0.00
26033400
26033600
26033800
26034000
position (bp)

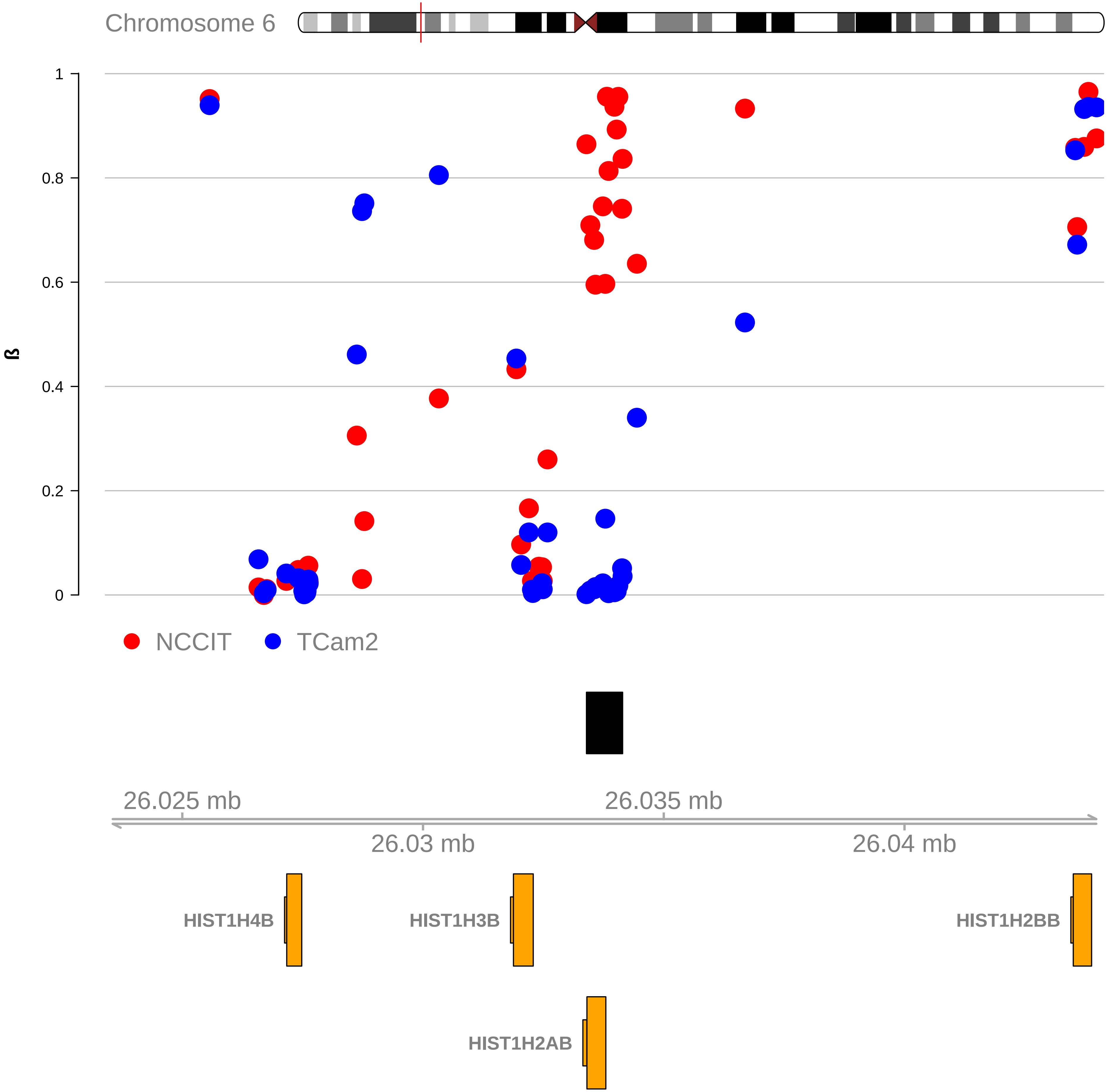

Chromosome 6
1
0.8
0.6
0.4
0.2
0
ß
NCCIT
TCam2
26.025 mb
26.035 mb
26.03 mb
26.04 mb
HIST1H4B
HIST1H3B
HIST1H2BB
HIST1H2AB
